# An Analysis of the Clinical Medication Rules of Traditional Chinese Medicine for Polycystic Ovary Syndrome Based on Data Mining

**DOI:** 10.1155/2023/6198001

**Published:** 2023-02-21

**Authors:** Dan Xu, Ming Lu, Ying Liu, Wansu Chen, Xiaole Yang, Min Xu, Huifang Zhou, Xiaoman Wei, Yao Zhu, Qingxia Song

**Affiliations:** ^1^Department of Gynecology, Suzhou TCM Hospital Affiliated to Nanjing University of Chinese Medicine, Suzhou 215009, China; ^2^Nanjing University of Chinese Medicine, Nanjing 210023, China; ^3^Nanjing Medical Data Mining Center, Nanjing 210023, China; ^4^Department of Gynecology, Affiliated Hospital of Nanjing University of Chinese Medicine, Nanjing 210029, China

## Abstract

**Objective:**

The aim of the present study is to investigate the rules and characteristics of the clinical administration of traditional Chinese medicine (TCM) in the treatment of polycystic ovary syndrome (PCOS) using data mining methods.

**Method:**

Medical cases of well-known contemporary TCM doctors treating PCOS were collected from the China National Knowledge Infrastructure, Chinese Biomedical Literature Service System, Wanfang, Chinese Scientific Journals Database, and PubMed; the data were then characterized, and a standardized database of medical cases was built. This database was used to (1) count the frequency of syndrome types and the herbs used in medical cases by data mining methods and (2) analyze drug association rules and systematic clustering methods.

**Results:**

A total of 330 papers were included, involving 382 patients and a total of 1,427 consultations. The most common syndrome type was kidney deficiency; sputum stasis was the core pathological product and causative factor. A total of 364 herbs were used. Among them, 22 herbs were used >300 times, including Danggui (*Angelicae Sinensis Radix*), Tusizi (*Semen Cuscutae*), Fuling (*Poria*), Xiangfu (*Nutgrass Galingale Rhizome*), and Baizhu (*Atractylodis Macrocephalae Rhizoma*). Additionally, 22 binomial associations were obtained from the analysis of association rules; five clustering formulae were obtained via the analysis of high-frequency drug clusters; and 27 core combinations were obtained by k-means clustering of formula.

**Conclusion:**

In the treatment of PCOS, TCM is primarily employed as a combination approach involving tonifying the kidneys, strengthening the spleen, eliminating damp and dissolving phlegm, activating blood circulation, and resolving blood stasis. The core prescription is primarily a compound intervention based on the Cangfu Daotan pill, Liuwei Dihuang pill, and Taohong Siwu decoction.

## 1. Introduction

Polycystic ovary syndrome (PCOS), a type of common gynecological disorder, affects approximately 8–13% of women within the reproductive age [1]. It is characterized by reproductive disorders, endocrine abnormalities, metabolic disorders, and mental health problems [2]; it is also one of the main causes of menstrual disorders and infertility in women and has developed into an area of focus in the field of gynecological endocrinology and reproductive medicine in recent years. Current treatment strategies for PCOS mainly include oral medications (short-acting combination oral contraceptives, progestins, insulin sensitizers, and ovulation induction), *in vitro* fertilization-embryo transfer, and surgery. However, long-term clinical practice suggests that the above-mentioned strategies have certain limitations, for example, high recurrence rates and invasiveness, unsatisfactory long-term results, and various side effects [3]. Recently, alternative therapies have been gradually coming into focus as a more favorable option for PCOS treatment [4, 5].

Traditional Chinese medicine (TCM) has unique advantages in the treatment of PCOS, and recent studies have shown that TCM can regulate the reproductive endocrine function of patients with PCOS as a whole, improve insulin resistance, promote ovulation, increase pregnancy rates, and improve various clinical symptoms; all with fewer adverse reactions and good safety [6–9]. However, due to the diverse clinical manifestations of PCOS and the complex nature of the disease, there is no unified or broadly recognized consensus on its diagnosis and treatment in the context of TCM.

The present paper deconstructs the TCM clinical differentiation and treatment rules of PCOS through systematic excavation and the study of modern medical cases involving TCM masters treating PCOS; this is of great significance for gaining a deeper understanding of the disease and improving the TCM differentiation and treatment system, as it concerns the condition.

## 2. Data and Methods

### 2.1. Data Sources

Five major medical databases (China National Knowledge Infrastructure, China Biology Medicine (CBM) disc, Wanfang, Chinese Scientific Journals Database, and PubMed) were searched using a computer. A combination of subject terms and basic searches was applied, and the search time limit ranged from the creation of each database to January 31, 2020.

**Figure 1:**
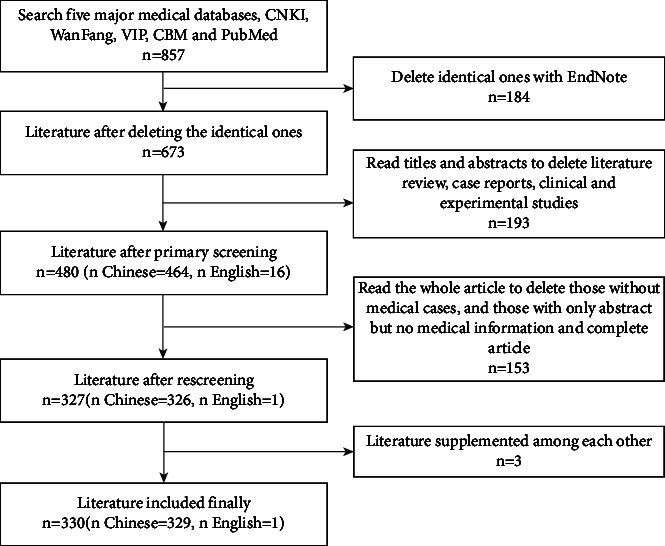
The flow chart of literature retrieval.

The Chinese searches included the following: (title: “Duonang Luancao Zonghezheng” or “PCOS”) and (title: “Yanan” or “Jingyan” or “Yanfang” or “Gean” or “Anli”). The English search terms were as follows: (“polycystic ovary syndrome” (Medical Subject Headings terms)) and (“Chinese phytotherapy” (Text Word)). A total of 857 relevant articles were retrieved. After removing repeated entries, initial screening entries, rescreening entries, and supplementary entries, a total of 330 articles (including 329 Chinese papers and 1 paper in English) were included according to the literature selection criteria. The specific retrieval and screening processes are shown in Figure 1.

### 2.2. Inclusion Criteria

The inclusion criteria for articles focusing on TCM cases were as follows: (1) academic experience, cases cited in medical treatises, and medical talks; (2) a Western medicine case diagnosis included PCOS; (3) the medical records of the first visit comprised symptoms, tongue and pulse, and prescriptions; and (4) if there were any changes in symptoms, syndrome type, treatment, prescription, or other aspects in a medical case between the first and second visit, the medical case of the second visit was recorded independently.

### 2.3. Exclusion Criteria

The exclusion criteria pertaining to case study articles were as follows: (1) literature reviews, case reports, animal experiments, and clinical studies; (2) cases lacking the necessary information (symptoms, lingual vessels, and prescriptions); (3) cases using acupuncture, auricular points, diet therapy, and ointment only for making a diagnosis and providing treatment; (4) for cases repeatedly published, only the first published medical case was retained; and (5) conference papers that included only abstracts but not the full text.

### 2.4. Diagnostic Criteria of PCOS

A confirmed diagnosis of PCOS was in accordance with the revised diagnostic criteria of PCOS set in the 2003 Rotterdam consensus workshop [10]: (1) sporadic ovulation or anovulation; (2) clinical symptoms (including hirsutism and/or acne) or biochemical evidence of hyperandrogenism (total testosterone >3.5 mmol/L); (3) polycystic ovarian lesions detected using the ultrasound method: ≥10–12 follicles with a diameter of 2–9 mm in each ovary and/or an ovarian volume of ≥l0 mL. Patients meeting either of the above criteria were diagnosed with PCOS.

### 2.5. Data Preprocessing and Normalization

A total of 330 articles, including 382 medical cases, and a total of 1,427 visits resulting in effective prescriptions were recorded using the Excel 2016 program to establish a PCOS prescription database. To ensure the accuracy of the data, the data were recorded independently by two people, and the results were checked after the recording was completed. The standardized TCM terminology was adopted by referring to *Zhongyi Linchuang Shuju Wajue Yanjiu Shuju Guifanhua Biaozhun* [11]. The syndrome types were standardized by referring to *zhongyi zhenduanxue* [12] (TCM Diagnostics) and *zhongyi fukexue* [13] (Gynecology of TCM), and the herbs were standardized through reference to the 2015 edition of *Zhonghua (Carthami Flos) Renmin Gongheguo Yaodian* [14], and *Zhongyaoxue* [15]. The following relevant examples were used: (1) the alias and common name of herbs, e.g., Qizi was classified as GouQizi (*Lycii Fructus*) and Xianlingpi as Yinyanghuo (*Epimedii Folium*); (2) herbs with a processing name or a name of origin, e.g., Quan Danggui and Danggui Shen were classified as Danggui (*Angelicae Sinensis Radix*), and Chuan Niuxi was classified as Niuxi (*Achyranthis Bidentatae Radix*). The data normalization process attempted to follow the original intention of the doctors involved in the cases.

### 2.6. The Data Analysis Platform

The data were analyzed using the data association analysis platform (XMiner v.1.0) of the Medcase V3.8 data recording and processing/mining system platform [16, 17], and the syndrome type and drug frequency of medical records were analyzed using frequency statistics. According to the number of prescriptions included and the prereading of relevant parameters, reasonable support and confidence values were set.

Association rule mining (an in-silico screening process) was applied to investigate the regularity of herbal compatibility in the prescriptions used in reported studies. The dataset and the association rules are defined as follows: an association rule has the form of left-hand side (LHS) ⇒ right-hand side (RHS), where LHS and RHS are sets of items. The occurrence of the RHS whenever the LHS set occurs is likely [18].

The Apriori algorithm was used to extract the significant associations from all possible combinations of items in the main dataset [19]. There were three evaluation metrics (support, confidence, and lift); these are critical in describing the power and significance of the rules generated by association rule mining [20]. Specifically, support is the frequency of the rule occurrence in the total dataset, measuring whether an association between the LHS and the RHS happens by chance. Confidence is the frequency of the rule occurrence in the cases of the dataset fulfilling the LHS of the rule, thus representing the reliability of the association. Lift is the ratio of observed support to the expected support when the LHS and the RHS are independent, indicating a dependency of the occurrences of the two items when its value is >1 [21]. Additionally, the association rule method was used to analyze the regularity of the herbal compatibility of the prescriptions and core herbs in the included medical records.

Next, the core combinations and new prescriptions were obtained by setting reasonable correlation and penalty degrees via the variable clustering method. In the present study, *k*-means cluster analysis was considered, as the variables were quantitative at the interval or ratio level rather than being either binary or counts. To avoid unreliable results through omitted variable bias, the authors of the present study enrolled all the attributes, including the meridian tropism, five properties, and five tastes and investigated the therapeutic preferences of the candidate clusters. Moreover, the authors compared the analysis results from different permutations of the initial center values to ensure an appropriate number of clusters; this was used to further assess the reliability of the given solution [22].

### 2.7. Analysis of Structural Diagram

The single item, single row, and single directional relationships in structural diagrams were shown as the green box, while the orange box represented a single item multirow, single directional, or single item multicolumn bidirectional relationship. The structural diagram of the associated rule sites in the drug sets can be taken as an example to explain the analysis rules in detail: the double row bidirectional relationship between the two drugs mostly represented that they are the common clinical drug pair; meanwhile, the double row bidirectional relationship existing among three drugs suggested that the three drugs were mostly clinical triplet herbs. Additionally, the single item and multirow bidirectional relationship between the two drugs mostly indicated that the two drugs appeared in clinical pairs in sequential chronological order, mainly manifesting as the drug emitted by the arrow appearing first and the drug pointed by the arrow appearing second. Moreover, the more times the drug was pointed by the arrow, the more times it was used in herbal compatibility. The analysis of the visual diagram for other item sets, such as symptoms and TCM pathology, was conducted with reference to the aforementioned drug sets.

## 3. Results

### 3.1. Frequency Statistics

#### 3.1.1. High-Frequency Syndrome Types

A total of 67 syndrome types were involved in 1,427 medical cases; among these, syndrome types with a frequency of >50 were related to phlegm stagnation, phlegm stasis, blood stasis stagnation, liver Qi stagnation, kidney deficiencies, and spleen and kidney deficiencies, accounting for 35.95% of the total clinical visits. The syndrome types with a frequency of >10 are shown in Table 1.

#### 3.1.2. High-Frequency Herbs

A total of 364 herb types were used in 1,427 medical cases; among these, 22 herbs were used ≥300 times (see Table 2). The top five herbs were Danggui (Angelicae Sinensis Radix), Tusizi (*Semen Cuscutae*), Fuling (*Poria*), Xiangfu (*Nutgrass Galingale Rhizome*), and Baizhu (*Atractylodis Macrocephalae Rhizoma*).

### 3.2. Association Rules

#### 3.2.1. Association Rules within a Syndrome Set

**Figure 2:**
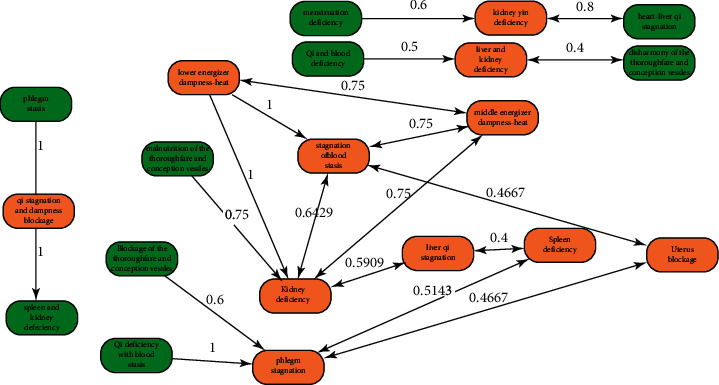
A structural diagram of the association rules' loci within the syndrome set. The apriori algorithm was used to analyze the association rules of the main syndromes of PCOS, applying settings in which support = 0.7%, confidence = 46%, and elevation > 1.

The association rule analysis of the main syndromes of PCOS was performed using the Apriori algorithm, applying settings in which support = 0.7%, confidence = 46%, and elevation >1. A total of 18 combinations of binomial association rules were obtained (see Table 3), and a structural chart of the association rules' loci concerning high-frequency evidence types was established (see Figure 2).

#### 3.2.2. Association Rules within the Herb Set

**Figure 3:**
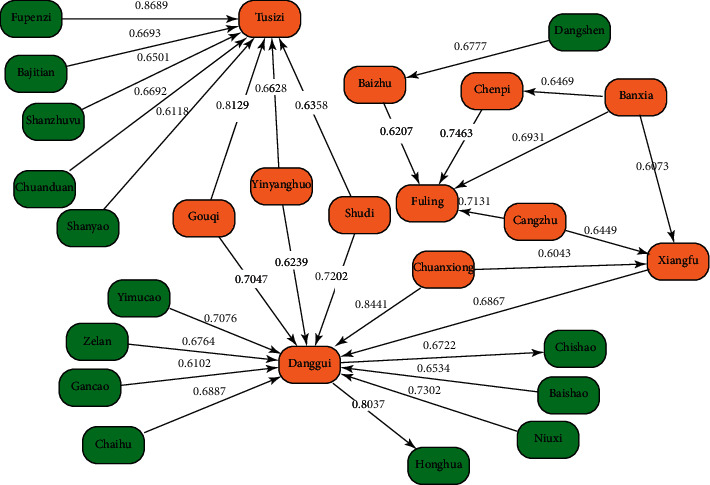
A structural diagram of the associated rule sites in the drug sets. The apriori algorithm was used to analyze the association rules between prescriptions and herbs in the TCM treatment of PCOS, applying settings in which support = 12%, confidence = 65% and lift measure > 1.

The Apriori algorithm was used to analyze the association rules between prescriptions and herbs in the TCM treatment of PCOS, using settings in which support = 12%, confidence = 65%, and lift measure >1. As a result, 22 binomial association rules were obtained (see Table 4) and employed to form the structural diagram of association rules' loci related to high-frequency herbs (see Figure 3).

### 3.3. Cluster Analysis

#### 3.3.1. High-Frequency Drug System Clustering Results

**Figure 4:**
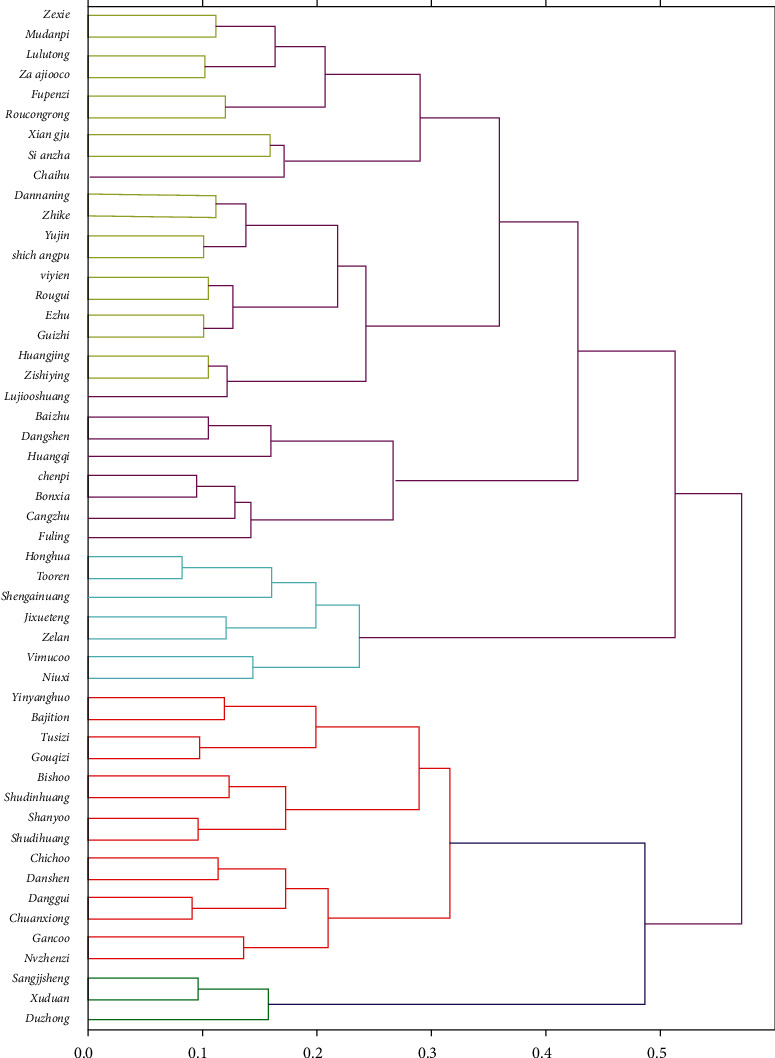
Tree diagram of the high-frequency drug cluster analysis: the green hierarchical clustering represents prescription 1, including Duzhong (*eucommiae cortex*), Chuanduan (*dipsaci radix*), and Sangjisheng (*taxilli herba*). The red hierarchical clustering represents prescription 2, including Nvzhenzi (*ligustri lucidi fructus*), Gancao (*glycyrrhizae radix et rhizoma*), Chuanxiong (*chuanxiong rhizoma*), Danggui (*angelicae sinensis radix*), Danshen (s*alviae miltiorrhizae radix et rhizoma*), Chishao (*paeoniae radix rubra*), Shanzhuyu (*corni fructus*), Shanyao (*dioscoreae rhizoma*), Shudihuang (*rehmanniae radix*), Baishao (*paeoniae radix alba*), GouQizi (*lycii fructus*), Tusizi (*semen cuscutae*), Bajitian (*morindae officinalis radix*), and Yinyanghuo (*epimedii folium*). The blue hierarchical clustering represents prescription 3, including Niuxi (*achyranthis bidentatae radix*), Yimucao (*leonuri herba*), Zelan (*eupatorium*), Jixueteng (*spatholobi caulis*), Shengdihuang (*rehmanniae radix*), Taoren (*persicae semen*), and Honghua (*carthami flos*). The purple hierarchical clustering represents prescription 4, including Fuling (*poria*), Cangzhu (*atractylodis rhizoma*), Banxia (*pinelliae rhizoma*), Chenpi (*citri reticulatae pericarpium*), HuangQi (*astragali radix*), Dangshen (*codonopsis radix*), and Baizhu (*atractylodis macrocephalae rhizoma*). The yellow hierarchical clustering represents prescription 5, including Lujiaoshuang (*cervi cornu degelatinatum*), Zishiying (*flourite fluoritum*), Huangjing (*polygonati rhizoma*), Guizhi (*cinnamomi ramulus*), Ezhu (*curcumae rhizoma*), Rougui (*cinnamomi cassiae cortex*), Yiyiren (*coicis semen*), Shichangpu (*acori tatarinowii rhizoma*), Yujin (*curcumae radix*), Zhike (*aurantii fructus*), Dannanxing (*arisaematis cum bile*), Chaihu (*Bupleurum chinense* DC.), Shanzha (*crataegi fructus*), Xiangfu (*nutgrass galingale rhizome*), Roucongrong (*Cistanche deserticola Ma*), Fupenzi (*rubi fructus*), Zaojiaoci (*gleditsia sinensis lam*), Lulutong (*fructus liquidambaris*), Mudanpi (*moutan cortex*), and Zexie (*alisma plantago-aquatica l*).

The systematic clustering method was applied to further analyze 51 high-frequency herbs (frequency ≥ 100) that were used to treat PCOS. Five clusters were selected from the clustering results and formulated into a cluster analysis tree diagram (see Figure 4). Meanwhile, the corresponding syndromes of each cluster prescription were summarized based on clinical experience (see Table 5).

#### 3.3.2. The *k*-Means Clustering Results of Prescription

In the drug cluster analysis, 27 clusters were present when *k*-value = 27.0000 and when inertia = 3310.6332 were used as a benchmark (see Table 6).

## 4. Discussion

The TCM context does not include the term “polycystic ovary syndrome”; however, according to the condition's clinical symptoms, it can be classified using the terms “amenorrhea,” “infertility,” “delayed menstruation,” and “scanty menstruation.” The “areolae” of “phlegm with blood stasis, reversible areolae” (as recorded in *Danxi Xinfa*) is presumed to be the earliest description of this disease in TCM.

Distinct from Western medicine, ancient books of TCM have recorded that the deficiencies of Qi, blood, yin, yang in Five Viscera (heart, liver, spleen, lung, and kidney) were involved in the pathogenesis development of PCOS. Proposed by TCM physicians, Qi is not only the energy of life but also the driving force behind the biological activities of the human body, mind, and spirit. Blood was the another vital TCM substance in charge of providing nutrients. The deficiencies or stagnant movement of Qi and blood will induce phlegm, dampness, and blood stasis, which stays and obstructs the uterus and consequently causes the blockage of the route of menstrual blood discharge and the polycystic-like pathologic changes of ovaries.

Additionally, according to TCM theory, kidney is the vital of Qi and blood, which produce essence and nourishes and maintains the physiological functions of the ovaries. Insufficient kidney essence induced declined and weakened function of ovaries, resulting in ovaries' dystrophy and various symptoms including failure to have a smooth menstrual flow owing to difficulty maturing or properly undischarged follicles. Similarly, the spleen provides nutrition for the body's Qi and blood production and circulation. Spleen insufficient induced the poor menstrual blood sources and difficultly draining menstrual blood due to the poor circulation of Qi and blood, leading to disordered menstrual cycle (the major symptom of PCOS). Additionally, liver Qi plays important role in the development of PCOS according to the theory of TCM. The stagnation of liver Qi means the poor circulation of liver Qi, which induced the accumulation of condensed pathological metabolites such as phlegm, dampness, and blood stasis, further leading to insufficient menstrual blood sources as well as difficultly draining menstrual blood.

Thus, it can be seen that “dysfunction of kidney, liver, and spleen” and “pathogenic metabolites such as phlegm, dampness, and blood stasis” interact with each other, resulting in PCOS [9]. This is consistent with the findings of the present study, in which the frequency analysis of high-frequency syndrome types shows that the common syndrome types of PCOS include phlegm stagnation, phlegm stasis, blood stasis stagnation, liver Qi stagnation, kidney deficiencies, and spleen and kidney deficiencies. Among these, kidney deficiency is the most important; this is in line with the classical theories, i.e., “menstruation comes from (the) kidney(s)” and “(the) kidney(s) (dominate) reproduction, which is the root of innate endowment.”

The results of the association rule analysis indicated a high correlation between Qi deficiency with blood stasis and phlegm stagnation, Qi stagnation, dampness blockage and phlegm stasis, lower energizer dampness-heat, and blood stasis stagnation. Phlegm and blood stasis are suggested as the most important pathological products and pathogenic factors of PCOS; this coincides with the theory of “phlegm mixed with blood stasis, reverse ke-nang,” proposed by Zhu Danxi.

It is worth noting that TCM with diverse bioactivities [23–26] has played a significant role in the treatment of refractory disease, such as PCOS. Therefore, the authors of the present study applied a frequency analysis to acquire the high-frequency herbs of treating PCOS in published clinical cases included in this study.

The results showed that the top 10 high-frequency herbs were Danggui (Angelicae Sinensis Radix), Tusizi (*Semen Cuscutae*), Fuling (*Poria*), Xiangfu (*Nutgrass Galingale Rhizome*), Baizhu (*Atractylodis Macrocephalae Rhizoma*), Danshen (*Salviae Miltiorrhizae Radix et Rhizoma*), Chuanxiong (*Chuanxiong Rhizoma*), Gancao (*Glycyrrhizae Radix et Rhizoma*), Shudihuang (*Rehmanniae Radix*), and Baishao (*Paeoniae Radix Alba*). Danggui (*Angelicae Sinensis Radix*), Chuanxiong (*Chuanxiong Rhizoma*), Shudihuang (*Rehmanniae Radix*), and Baishao (*Paeoniae Radix Alba*) were used to tonify the essence and blood as well as to activate the blood to regulate menstruation.

Tusizi (*Semen Cuscutae*) was used to reinforce kidney essence and warm the kidney yang. Fuling (*Poria*), Baizhu (*Atractylodis Macrocephalae Rhizoma*), and Gancao (*Glycyrrhizae Radix et Rhizoma*) were used to fortify the spleen, replenish Qi, and drain dampness. Xiangfu (*Nutgrass Galingale Rhizome*) was used to soothe the liver, regulate Qi, and help resolve depression. Danshen (*Salviae Miltiorrhizae Radix et Rhizoma*) was applied to activate the blood and resolve its stasis as well as regulate menstruation. This corresponds with the pathogenesis of deficiency, dampness, and stasis. It is also consistent with the physiological characteristics “blood is the base of the female,” “kidneys dominate reproduction, and the spleen controls digestion, which are considered the root cause of innate endowment and postnatal constitution.” Additionally, the results of pharmacological experiments further validated that the aforementioned herbs could perform protective effects on PCOS through multiple pathways. For example, a report has shown that aqueous extract from Danggui (*Angelicae Sinensis Radix*) had a beneficial effect on a rat with PCOS and that the underlying mechanism was partly related to the JAK2/STAT3 signaling pathway mediated by interleukin-6 [27]. The total flavone of Tusizi (*Semen Cuscutae*) could improve PCOS by regulating the secretion of estrogen and androgen as well as affect the hypothalamic-pituitary-ovary axis pathway [28].

The results of the association rules in the drug sets showed that drug combinations with a confidence level of >70% included the following: Fupenzi (*Rubi Fructus*) ⟶ Tusizi (*Semen Cuscutae*), Chuanxiong (*Chuanxiong Rhizoma*) ⟶ Danggui (*Angelicae Sinensis Radi*x), GouQizi (*Lycii Fructus*) ⟶ Tusizi (*Semen Cuscutae*), Honghua (*Carthami Flos*) ⟶ Danggui (*Angelicae Sinensis Radix*), Chenpi (*Citri Reticulatae Pericarpium*) ⟶ Fuling (*Poria*), Niuxi (*Achyranthis Bidentatae Radix*) ⟶ Danggui (*Angelicae Sinensis Radix*), Shudihuang (*Rehmanniae Radix*) ⟶ Danggui (*Angelicae Sinensis Radix*), Cangzhu (*Atractylodis Rhizoma*) ⟶ Fuling (*Poria*), Yimucao (*Leonuri Herba*) ⟶ Danggui (*Angelicae Sinensis Radix*), and GouQizi (*Lycii Fructus*) ⟶ Danggui (*Angelicae Sinensis Radix*); 6 of the above 10 association rules involved Danggui (*Angelicae Sinensis Radix*), suggesting that it was the most commonly used herb in cases of PCOS. This is consistent with the results related to the drug frequency distribution. Danggui (*Angelicae Sinensis Radix*) is a key medicine for nourishing and activating the blood as well as for regulating menstruation. According to *Jingyue quanshu·bencaozheng*, “the taste is sweet and heavy, so it can nourish the blood, (and) its (smell is) light and pungent, so it can move blood (…) there is moving in the nourishing, nourishing in the moving, as the Qi medicine in the blood, as well as the panacea medicine in the blood.”

A total of 5 clustered prescriptions were obtained by systematic clustering. According to the syndrome type inferred from the prescription, clustering prescription 1 has the function of reinforcing the liver and kidneys and warming the kidney yang, which is suitable for kidney yang deficiency syndrome. Clustering prescription 2 has the function of nourishing the kidney yin and nourishing and activating the blood, which is suitable for blood stasis in the case of kidney-deficiency syndrome. Clustering prescription 3 has the function of activating and resolving blood stasis and tonifying the blood to regulate menstruation, which is suitable for blood stasis and amenorrhea syndrome. Clustering prescription 4 has the function of fortifying the spleen and replenishing the Qi as well as drying dampness to resolve phlegm, which is suitable for a spleen deficiency with phlegm and dampness syndrome. Clustering prescription 5 has the function of warming the yang and resolving phlegm as well as resolving stasis and dredging collaterals, which is suitable for kidney deficiency with phlegm stasis syndrome.

The *k*-means clustering analysis of the formulations yielded 27 types of core prescriptions. Among them, clustering prescriptions 1 and 13 both include Cangzhu (Atractylodis Rhizoma), Fuling (*Poria*), Chenpi (*Citri Reticulatae Pericarpium*), and Xiangfu (*Nutgrass Galingale Rhizome*); the main effects are strengthening the spleen, drying dampness, and resolving phlegm as well as treating spleen deficiencies with dampness encumbrance syndrome, which is presumed to be the addition and subtraction of prescription of the Cangfu Daotan pill according to the drug composition.

Most patients with PCOS are obese, as per *Zhulin nvke zhengzhi*: “the body hypertrophy has phlegm and Qi deficiency, to a few months to begin menstruation, appropriate to take shape more phlegm deficiency, to several months and the originator, appropriate to take Cangfu Liujun decoction, as well as Cangfu Daotan pill.” Clustering prescription 7 includes Banxia (*Pinelliae Rhizoma*), Dannanxing (*Arisaematis Cum Bile*), Chenpi (*Citri Reticulatae Pericarpium*), and Xiangfu (*Nutgrass Galingale Rhizome*), and its main efficacy relates to dampness and resolving phlegm; accordingly, it is useful in the treatment of phlegm stagnation syndrome.

According to the drug composition of the prescription, it is speculated that the addition and subtraction prescription of the Erchen decoction. Clustering prescriptions 15 and 20 both include Mudanpi (*Moutan Cortex*), Shanzhuyu (*Corni Fructus*), Shanyao (*Dioscoreae Rhizoma*), Chuanxiong (*Chuanxiong Rhizoma*), and Shudihuang (*Rehmanniae Radix*). Their main efficacy is nourishing the kidney yin; hence, they are used in the treatment of kidney yin deficiency syndrome. According to the prescription source knowledge, it is speculated that they are the addition and subtraction prescriptions of the Liuwei Dihuang pill. Clustering prescriptions 3 and 26 include Danggui (*Angelicae Sinensis Radix*), Zhishi (*Citrus aurantium* L), Niuxi (*Achyranthis Bidentatae Radix*), Taoren (*Persicae Semen*), and Honghua (*Carthami Flos*). Their primary efficacy relates to activating and resolving blood stasis; as such, they are used in the treatment of blood stasis stagnation. According to the prescription source knowledge, it is speculated that they are the addition and subtraction prescriptions of the Taohong Siwu decoction.

## 5. Conclusion

The present data mining research analyzed published medical cases of PCOS that were derived from the findings of well-known TCM doctors to explore the experiences of PCOS syndrome types, treatment methods, and medications by different analysis methods. The results showed that the common syndrome types include phlegm stagnation, phlegm stasis, blood stasis stagnation, liver Qi stagnation, kidney deficiencies, and spleen and kidney deficiencies; among these, kidney deficiency is the core syndrome type, and phlegm and blood stasis are the core pathological products and pathogenic factors. Commonly used medicines for treating these conditions include Danggui (*Angelicae Sinensis Radix*), Tusizi (*Semen Cuscutae*), Fuling (*Poria*), Xiangfu (*Nutgrass Galingale Rhizome*), Baizhu (*Atractylodis Macrocephalae Rhizoma*), Danshen (*Salviae Miltiorrhizae Radix et Rhizoma*), Chuanxiong (*Chuanxiong Rhizoma*), and Gancao (*Glycyrrhizae Radix et Rhizoma*). The main efficacy of high-frequency drug system clustering prescription includes reinforcing the liver and kidney, warming the kidney yang, nourishing the kidney yin, nourishing and activating the blood, activating and resolving blood stasis, tonifying the blood to regulate menstruation, fortifying the spleen and replenishing the Qi, drying dampness to resolve phlegm, warming the yang and resolving phlegm, resolving stasis, and dredging collaterals. The treatment methods that are typically used apply a combination of compound methods, such as tonifying the kidneys, strengthening the spleen, resolving dampness, activating the blood, and resolving stasis. Most of the core prescriptions were TCM compound formula interventions based primarily on the combination of the Cangfu Daotan pill, Liuwei Dihuang pill, and Taohong Siwu decoction.

Therefore, this study will provide guiding significance for the formation of a consensus on the clinical treatment of PCOS by TCM experts in terms of its diagnosis and treatment as well as the relevant medication use.

## Figures and Tables

**Table 1:** Distribution of syndrome type (frequency ≥ 10).

Sequence	Syndrome type	Time	Frequency (%)
1	Kidney deficiency	135	9.46
2	Phlegm stagnation	100	7.01
3	Phlegm stasis	74	5.19
4	Stagnation of blood stasis	72	5.05
5	Liver Qi stagnation	66	4.63
6	Spleen and kidney deficiency	66	4.63
7	Spleen deficiency	35	2.45
8	Kidney yang deficiency	22	1.54
9	Liver and kidney deficiency	20	1.40
10	Uterus blockage	15	1.05

**Table 2:** High-frequency herb distribution (frequency ≥ 300).

Sequence	Herb	Time	Frequency (%)	Sequence	Herb	Time	Frequency (%)
1	Danggui	800	56.06	12	Yinyanghuo	437	30.62
2	Tusizi	710	49.75	13	Chenpi	413	28.94
3	Fuling	659	46.18	14	Shanyao	407	28.52
4	Xiangfu	619	43.38	15	Chuanduan	393	27.54
5	Baizhu	554	38.82	16	Shanzhuyu	363	25.44
6	Danshen	550	38.54	17	Yimucao	358	25.09
7	Chuanxiong	513	35.95	18	Cangzhu	352	24.67
8	Gancao	508	35.60	19	GouQizi	344	24.11
9	Shudihuang	496	34.76	20	HuangQi	306	21.44
10	Baishao	478	33.50	21	Banxia	303	21.23
11	Niuxi	440	30.83	22	Chisaho	300	21.02

**Table 3:** Set of association rule items within the syndrome set.

Sequence	Rule itemset	Support (%)	Confidence (%)	Lift
1	Uterus blockage ⟶ stagnation of blood stasis	1.85	46.67	2.53
2	Malnutrition of the thoroughfare and conception vessels ⟶ kidney deficiency	0.79	75.00	2.11
3	Blockage of the thoroughfare and conception vessels ⟶ phlegm stagnation	0.79	60.00	2.25
4	Liver Qi stagnation ⟶ kidney deficiency	10.29	59.09	1.66
5	Menstruation deficiency ⟶ kidney yin deficiency	0.79	60.00	18.95
6	Spleen deficiency ⟶ phlegm stagnation	4.75	51.43	1.91
7	Qi deficiency with blood stasis ⟶ phlegm stasis	0.79	100.00	3.75
8	Qi and blood deficiency ⟶ liver and kidney deficiency	0.79	50.00	9.48
9	Qi stagnation and dampness and dampness blockage ⟶ spleen and kidney deficiency	0.79	100.00	5.74
10	Qi stagnation and dampness blockage ⟶ phlegm stasis	0.79	100.00	5.12
11	Lower energizer dampness-heat ⟶ middle energizer dampness-heat	0.79	100.00	94.75
12	Lower energizer dampness-heat ⟶ stagnation of blood stasis	0.79	100.00	5.41
13	Lower energizer dampness-heat ⟶ kidney deficiency	0.79	100.00	2.81
14	Heart-liver Qi stagnation ⟶ kidney yin deficiency	1.06	80.00	25.27
15	Stagnation of blood stasis ⟶ kidney deficiency	11.87	64.29	1.80
16	Middle energizer dampness-heat ⟶ lower energizer dampness-heat	0.79	75.00	94.75
17	Middle energizer dampness-heat ⟶ stagnation of blood stasis	0.79	75.00	4.06
18	Middle energizer dampness-heat ⟶ kidney deficiency	0.79	75.00	2.11

^*∗*^ *Note.* Support ≥ 0.7%; confidence ≥ 46%; lift measure > 1.

**Table 4:** Set of associated rule items within the drug set.

Sequence	Rule itemset	Support (%)	Confidence (%)	Lift
1	Bajitian ⟶ Tusizi	12.17	66.93	1.34
2	Baishao ⟶ Danggui	22.26	65.34	1.14
3	Banxia ⟶ Fuling	15.03	69.31	1.50
4	Banxia ⟶ Chenpi	14.03	64.69	2.20
5	Cangzhu ⟶ Fuling	17.97	71.31	1.54
6	Chaihu ⟶ Danggui	12.67	68.87	1.20
7	Chen pi ⟶ Fuling	21.90	74.63	1.61
8	Chishao ⟶ Danggui	14.39	67.22	1.18
9	Chuanduan ⟶ Tusizi	18.68	66.92	1.34
10	Chuanxiong ⟶ Danggui	30.99	84.41	1.48
11	Dangshen ⟶ Baizhu	13.24	67.77	1.72
12	Fupenzi ⟶ Tusizi	12.81	86.89	1.74
13	GouQizi ⟶ Tusizi	19.90	81.29	1.64
14	GouQizi ⟶ Danggui	17.25	70.47	1.23
15	Honghua ⟶ Danggui	12.31	80.37	1.41
16	Niuxi ⟶ Danggui	22.48	73.02	1.28
17	Shanzhuyu ⟶ Tusizi	16.89	65.01	1.30
18	Shudihuang ⟶ Danggui	25.05	72.02	1.26
19	Xiangfu ⟶ Danggui	30.28	68.67	1.20
20	Yimucao ⟶ Danggui	17.32	70.76	1.24
21	Yinyanghuo ⟶ Tusizi	20.69	66.28	1.32
22	Zelan ⟶ Danggui	13.31	67.64	1.18

^*∗*^ *Note.* Support ≥ 12%; confidence ≥ 65%; lift measure > 1.

**Table 5:** High-frequency drug system clustering (frequency range > 100).

Classification	Clustering prescription	Effect	Indicated syndrome
1st	Duzhong, Chuanduan, Sangjisheng	Tonify the liver and warm the kidney	Kidney yang deficiency
2nd	Nvzhenzi, Gancao, Chuanxiong, Danggui, Danshen, Chishao, Shanzhuyu, Shanyao, Shudihuang, Baishao, Gouqizi, Tusizi, Bajitian, Yinyanghuo	Enrich the kidney yin, tonify and activate blood	Kidney deficiency and blood stasis
3rd	Niuxi, Yimucao, Zelan, Jixueteng, Shengdihuang, Taoren, Honghua	Activate blood, remove stasis, nourish blood and regulate menstruation	Amenorrhea due to blood stasis
4th	Fuling, Cangzhu, Banxia, Chenpi, HuangQi, Dangshen, Baizhu	Strengthen the spleen, invigorate Qi, dry dampness and transform phlegm	Spleen deficiency with phlegm dampness
5th	Lujiaoshuang, Zishiying, Huangjing, Guizhi, Ezhu, Rougui, Yiyiren, Shichangpu, Yujin, Zhike, Dannanxing, Chaihu, Shanzha, Xiangfu, Roucongrong, Fupenzi, Zaojiaoci, Lulutong, Danpi, Zexie	Warm yang, transform phlegm, eliminate stasis and unblock collateral	Kidney deficiency with phlegm and stasis

**Table 6:** *K*-means clustering of prescription.

Classification	Site number (site value)	Clustering prescription
1^st^	18 (40 116 117 118 119 325 327 330 633 891 892 893 894 895 1044 1045 1046 1207)	Cangzhu, Fuling, Chenpi, Xiangfu
2^nd^	13 (487 488 489 490 491 492 493 494 495 498 499 500 501)	Chuanduan, Sangjisheng, Zishiying, Gusuibu
3^rd^	12 (707 708 709 710 711 712 713 714 715 716 717 718)	Danggui, Zhishi, Niuxi, Gancao, Shengdihuang, Zhimu, Dannanxing, Chenpi, Xiangfu, Huangbai
4^th^	11 (31 44 521 522 523 524 525 526 527 615 1086)	Chaihu, Baishao, Tusizi, Chishao
5^th^	9 (11 12 13 14 15 16 17 753 1304)	Mohanlian, Nvzhenzi, Shanzhuyu, Shanyao, Sangshen
6^th^	9 (228 229 230 429 446 447 448 459 460)	Danshen, Sangye, Zishiying, Huangbai
7^th^	9 (625 626 627 799 1264 1266 1268 1367 1368)	Banxia, Dannanxing, Chenpi, Xiangfu
8^th^	8 (1099 1100 1101 1102 1103 1104 1105 1106)	Danshen, Danpi, Dahuang, Pipaye, Chaihu, Zhizi, Niuxi, Shengdihuang, Shenqu, Zicao, Qiancao, Guya, Xiangfu, Maiya, Longdancao
9^th^	8 (687 689 691 692 694 696 698 699)	Danpi, Shanzhuyu, Shanyao, Guizhi, Sangye, Zexie, Shengdihuang, Zhuru, Fuling, Fuzi, Longgu
10^th^	8 (819 821 822 823 824 825 826 827)	SanQi, Daxueteng, Chaihu, Zexie, Zhimu, Xiangfu, Huangbai
11^st^	8 (1024 1025 1026 1027 1028 1029 1152 1206)	Dangshen, Banxia, Shanzha, Baizhu, Fuling, Jixueteng
12^nd^	8 (768 770 771 772 773 775 777 778)	Danshen, Shengdihuang, Baizhu, Fuling, Tusizi
13^rd^	8 (624 796 797 798 1216 1217 1218 1219)	Banxia, Cangzhu, Fuling, Chenpi, Xiangfu
14^th^	8 (1192 1193 1194 1195 1196 1197 1245 1246)	Difuzi, Baixianpi, Fuling, Chenpi, Xiangfu
15^th^	7 (752 754 755 756 757 1315 1316)	Danpi. Shanzhuyu, Shanyao, Chuangxiong, Danggui, Shudihuang, Niuxi, Gancao, Honghua
16^th^	7 (81 830 831 832 1107 1108 1109)	Zelan, Zexie, Shudihuang, Lizhihe, Lulutong
17^th^	7 (997 1072 1075 1354 1355 1356 1357)	Danggui, Yinyanghuo, Niuxi, Zishiying, Tusizi
18^th^	7 (763 928 929 930 931 932 1121)	Chaihu, Guizhi, Chishao, Jinyinhua
19^th^	7 (357 358 359 360 361 362 372)	Shanyao, Danggui, Baizhu, Xiangfu
20^th^	6 (386 1002 1003 1004 1005 1006)	Danpi, Shanzhuyu, Shanyao, Chuanxiong, Shudihuang, Shengdihuang, Baishao, Fuling, Chishao, Xiangfu
21^st^	6 (853 854 855 856 857 1379)	Banxia, Chuanxiong, Baizhu, Shenqu, Xiangfu
22^nd^	6 (393 394 395 396 397 398)	Xianmao, Dangshen, Shichangpu, Yuanzhi, Xiangfu
23^rd^	5 (3 4 5 6 7)	Juemingzi, Shanzha, Shanyao, Guizhi, Sangye, Gancao, Cangzhu, Fuling, Heye, Gegen, Yiyiren, Maiya, Huangjing
24^th^	5 (8 9 10 18 19)	Heshouwu, Dongguapi, Juemingzi, Shanyao, Zelan, Shichangpu, Cangzhu, Heye, HuangQi
25^th^	5 (517 518 519 520 1327)	Dangshen, Shengma, Danggui, Chaihu, Gancao, Baizhu, Chenpi, HuangQi
26^th^	5 (1231 1232 1233 1234 1381)	Zhike, Taoren, Gaocao, Baishao, Honghua, Fuling, Jineijin
27^th^	4 (881 882 883 884)	Danshen, Chuanduan, Chuanlianzi, Chuanxiong, Danggui, Duzhong, GouQizi, Niuxi, Ezhu, Tusizi, Ejiao, Biejia, Maiya, HuangQi

## Data Availability

The data that support the findings of this study are available from the corresponding author upon reasonable request.
